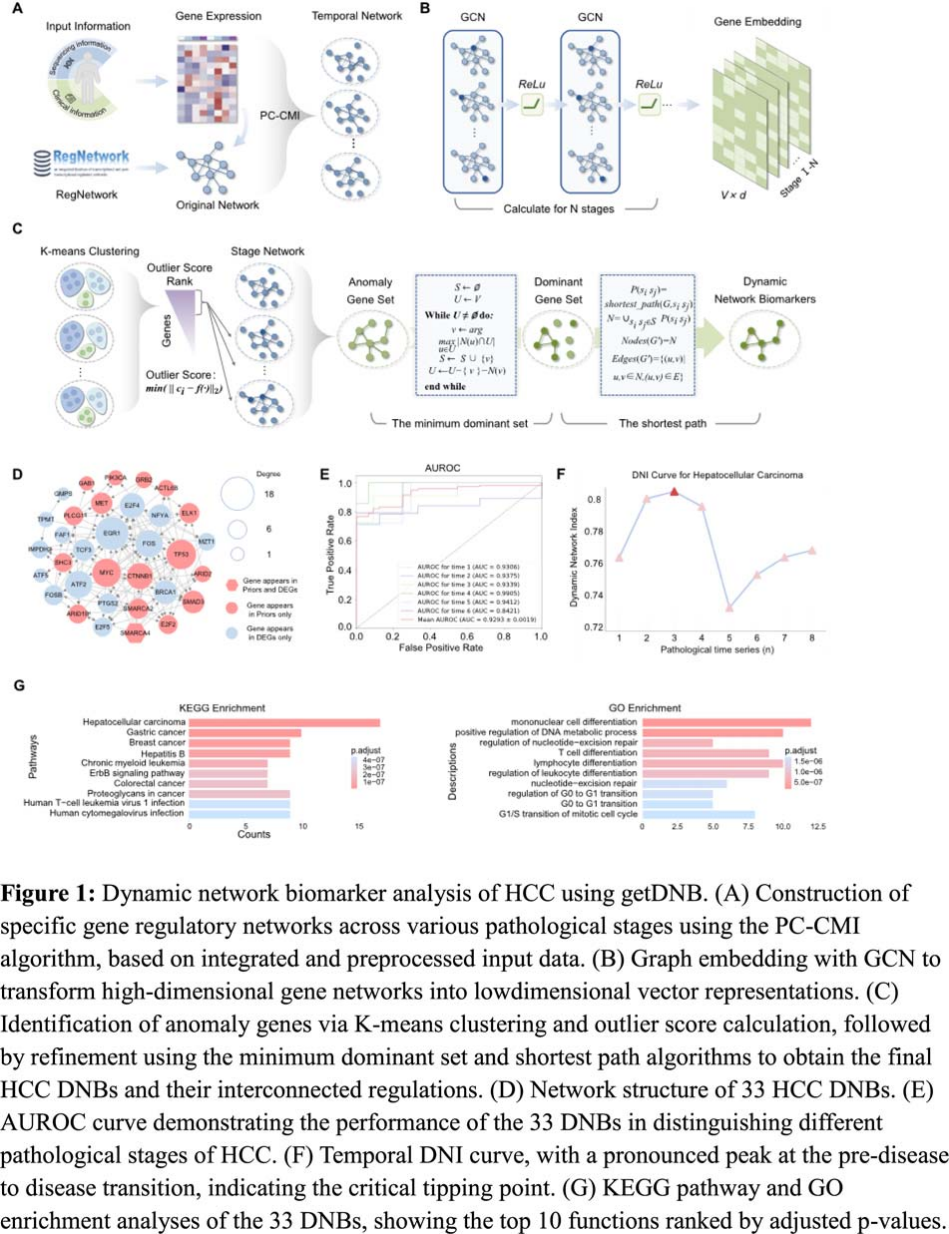# getDNB: identifying dynamic network biomarkers from time-varying gene regulations utilizing graph embedding techniques

**DOI:** 10.1093/bib/bbaf631.009

**Published:** 2025-12-12

**Authors:** Tong Wang, Lingyu Li, Zhi-Ping Liu

**Affiliations:** Department of Biomedical Engineering, School of Control Science and Engineering, Shandong University, Jinan, Shandong 250061, China; School of Biomedical Sciences, The University of Hong Kong, Hong Kong SAR, China; Department of Biomedical Engineering, School of Control Science and Engineering, Shandong University, Jinan, Shandong 250061, China

## Abstract

**Aim:**

Complex diseases remain difficult to detect early because conventional diagnostic strategies rely on static biomarkers that typically emerge at advanced stages. We aimed to develop a computational framework to systematically identify dynamic network biomarkers (DNBs) from temporally evolving gene regulatory networks.

**Methods:**

We present getDNB, a graph embedding technique framework with three main steps: (1) constructing stage-specific regulatory networks to capture dynamic alterations in molecular interactions during disease progression; (2) employing graph convolutional networks (GCN) to project these high-dimensional networks into low-dimensional embeddings while preserving topological structure; (3) quantifying gene-level abnormality via K-means clustering and outlier scores, followed by network refinement using minimum dominating set and shortest path algorithms to ensure network connectivity and reduce redundancy. Additionally, a dynamic network index (DNI) was introduced to quantify temporal fluctuations in network disorder, providing a quantitative signal for critical transition states.

**Results:**

Applied to a hepatocellular carcinoma (HCC) dataset, getDNB identified 33 robust DNBs and their interconnected network, achieving high predictive accuracy (AUROC = 0.929). The DNI curve exhibited a pronounced increase at the pre-disease stage, consistent with complex system transition theory predictions. Functional enrichment analysis revealed significant associations of these DNBs with key oncogenic pathways, including hepatocellular carcinoma, hepatitis B infection, and cell cycle regulation.

**Conclusion:**

getDNB provides a powerful and generalizable approach for dynamic biomarker discovery. By integrating graph neural networks, anomaly detection, and network optimization, it offers mechanistic insights into complex disease progression and enables identification of early-warning signals with potential clinical translational value.

**References:**

1. Chen L, Liu R, Liu Z, Li M, Aihara K. ‘Detecting early-warning signals for sudden deterioration of complex diseases by dynamical network biomarkers.’ Scientific Reports 2012; 2: 342.

2. Wang T, Liu Z. ‘getDNB: identifying dynamic network biomarkers of hepatocellular carcinoma from time-varying gene regulations utilizing graph embedding techniques for anomaly detection.’ Bioinformatics 2025; 41(9): btaf518.